# Cross-border malaria drivers and risk factors on the Brazil–Venezuela border between 2016 and 2018

**DOI:** 10.1038/s41598-022-09819-0

**Published:** 2022-04-11

**Authors:** Kinley Wangdi, Erica Wetzler, Paola Marchesini, Leopoldo Villegas, Sara Canavati

**Affiliations:** 1 Department of Global Health, National Centre for Epidemiology and Population Health, College of Health and Medicine, The Australian National University, Canberra, ACT 2601 Australia; 2 World Vision US, 34834 Weyerhaeuser Way South, Federal Way, Washington, USA; 3 Malaria Technical Group, Department of Surveillance for Zoonotic and Vector Borne Diseases, Ministry of Health, Brasilia, Federal District Brazil; 4 Global Development One, Silver Spring, MD USA; 5 Asociación Civil Impacto Social (ASOCIS), Tumeremo, Bolívar Venezuela

**Keywords:** Diseases, Risk factors

## Abstract

Globally, cross-border importation of malaria has become a challenge to malaria elimination. The border areas between Brazil and Venezuela have experienced high numbers of imported cases due to increased population movement and migration out of Venezuela. This study aimed to identify risk factors for imported malaria and delineate imported malaria hotspots in Roraima, Brazil and Bolivar, Venezuela between 2016 and 2018. Data on malaria surveillance cases from Roraima, Brazil and Bolivar, Venezuela from 2016 to 2018 were obtained from national surveillance systems: the Brazilian Malaria Epidemiology Surveillance Information System (SIVEP-Malaria), the Venezuelan Ministry of Health and other non-government organizations. A multivariable logistic regression model was used to identify the risk factors for imported malaria. Spatial autocorrelation in malaria incidence was explored using Getis-Ord (Gi*) statistics. During the study period, there were 11,270 (24.3%) and 4072 (0.7%) imported malaria cases in Roraima, Brazil and Bolivar, Venezuela, respectively. In the multivariable logistic regression for Roraima, men were 28% less likely to be an imported case compared to women (Adjusted Odds Ratio [AOR] = 0.72; 95% confidence interval [CI] 0.665, 0.781). Ages 20–29 and 30–39 were 90% (AOR = 1.90; 95% CI 1.649, 2.181) and 54% (AOR = 1.54; 95% CI 1.331, 1.782) more likely to be an imported case compared to the 0–9 year age group, respectively. Imported cases were 197 times (AOR = 197.03; 95% CI 175.094, 221.712) more likely to occur in miners than those working in agriculture and domestic work. In Bolivar, cases aged 10–19 (AOR = 1.75; 95% CI 1.389, 2.192), 20–29 (AOR = 2.48; 95% CI 1.957, 3.144), and 30–39 (AOR = 2.29; 95% CI 1.803, 2.913) were at higher risk of being an imported case than those in the 0–9 year old group, with older age groups having a slightly higher risk compared to Roraima. Compared to agriculture and domestic workers, tourism, timber and fishing workers (AOR = 6.38; 95% CI 4.393, 9.254) and miners (AOR = 7.03; 95% CI 4.903, 10.092) were between six and seven times more likely to be an imported case. Spatial analysis showed the risk was higher along the international border in the municipalities of Roraima, Brazil. To achieve malaria elimination, cross-border populations in the hotspot municipalities will need targeted intervention strategies tailored to occupation, age and mobility status. Furthermore, all stakeholders, including implementers, policymakers, and donors, should support and explore the introduction of novel approaches to address these hard-to-reach populations with the most cost-effective interventions.

## Introduction

Recognizing the need to hasten progress in reducing the burden of malaria, the World Health Organization (WHO) developed the *Global Technical Strategy for Malaria 2016–2030* (GTS)^1^, which sets out a vision for accelerating progress towards malaria elimination. The WHO strategy is complemented by the Roll Back Malaria *Action and Investment to Defeat Malaria 2016–2030* (AIM)^2^. GTS and AIM set an ambitious global target of eliminating malaria in at least 21 countries by 2020, known as E-2020 countries and 35 countries by 2030^2^. In the WHO Region of the Americas, reported malaria cases reduced by 40% (from 1.5 million to 0.9 million cases) and incident cases by 57% from 2000 to 2019^3^. As a result, 17 countries plus one territory in the Americas are aiming for malaria elimination^4^. Despite the success in malaria control, the region’s progress has stalled in recent years due to the major surge in malaria in Venezuela, which increased from 35,500 cases in 2000 to over 467,000 by 2019^3^.

Globally, cross-border malaria poses significant challenges to malaria elimination efforts^5–7^. The relevance of cross-border malaria is more pronounced in countries where there is transmission differential across the border due to a gradient in receptivity or intervention coverage^7^. Cross-border malaria is a complex and multi-faceted issue, with a number of reasons for continuing transmission^7^. For example, border areas are often remote, less developed, sometimes more forested and likely to have weak health systems^8^. Globally, surveillance is reported to be low and insufficient to cover mobile and migrant populations, nomadic and indigenous populations, or minorities who normally gather or reside along borders^9,10^. Migration, an important driver of cross-border malaria, occurs as people search for better economic, work and social opportunities^11–15^. Furthermore, political unrest, climate change, and conflict leads to increased cross-border malaria due to migration^16^. Also some indigenous populations tend to have nomadic lifestyles, routinely crossing borders over time.

The unstable political, social, and economic situation in Venezuela has led to increased migration to neighboring countries including Brazil and Guyana^17^. Along the Peru and Ecuador international borders, considerable spillover of imported malaria cases from Venezuelan migrants is likely to derail elimination efforts in those countries^18,19^. Further, unregulated mining, particularly in Bolívar state, has been linked to the rapid increase and spread of malaria cases to other regions of the country^20^.

Therefore, identifying hotspots of cross-border malaria along international borders is useful for improved surveillance through cross-country collaborations^21,22^. In addition, identification of malaria hotspots in certain areas can help to detect problem areas, which can be further investigated to pinpoint possible causes of relatively higher incidences of malaria in a particular area^23^. Hotspots analysis can be done through spatial epidemiological tools (including Geographical Information Systems [GIS] and spatial analytic methods)^11,24,25^. Similarly, understanding the socio-demographic profiles of cross-border malaria is imperative for designing locally appropriate prevention measures and operational plans. Examples of such operational exercises include provision of pre-departure and on-the-way education at screening posts^26,27^.

**Figure 1:**
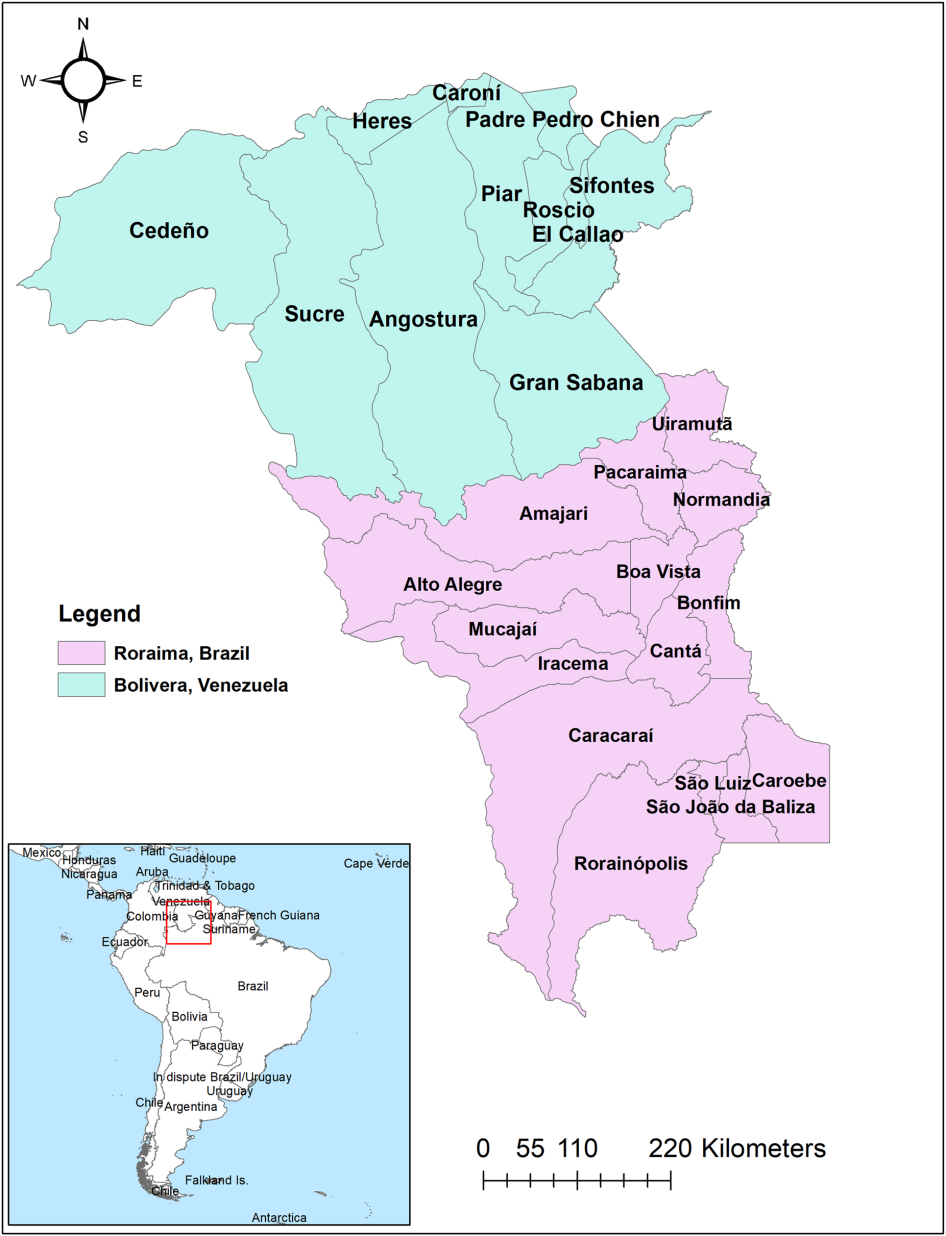
Map of Roraima State, Brazil and Bolivar State, Venezuela.

In this study, we aimed to identify the risk factors and map the hotspots of imported malaria in Bolívar state, Venezuela and Roraima state, Brazil between 2016 and 2018 (Fig. 1).

## Results

### Descriptive analysis

A total of 46,420 and 584,715 malaria cases were reported in Roraima and Bolivar states respectively during the study period (2016–2018). Of these cases, 11,270 (24.3%) and 4072 (0.7%) were imported cases from other countries, respectively. In Roraima, 63.5% (7360) and in Bolivar, 70.5% (2870) of imported cases were among males, and most of them were in the age group of 20–29 years in both states: 31.1% (3459) and 39.5% (1608) in Roraima and Bolivar, respectively. More than 78.1% (8689) of imported cases occurred among mining populations in Roraima state, and 63.0% (2564) in Bolivar state. In both Roraima and Bolivar states, 94.1% (10,607) and 90.8% (3696) of imported cases were found among non-indigenous people. *Plasmodium vivax* infections made up 69.2% (7796) and 72.5% (2953) of imported cases in Roraima and Bolivar states, respectively. Venezuela (82.3%, 9270), Guyana (17.4%, 1957) and other countries (0.4%, 43) were the origin of imported reported in Roraima. In Bolivar, imported cases from Guyana were 98.9% (4016), while Brazil and other countries were 1.2% (50) and 0.2% (6), respectively (Table 1).

**Table 1:** Socio-demographic characteristics of total and imported malaria cases in Roraima, Brazil and Bolivar, Venezuela: 2016–2018.

Characteristic		Roraima, Brazil		Bolivar, Venezuela	
		Total (%)	Imported (%)	Total (%)	Imported (%)
**Sex**					
Women		17,101 (36.8)	3910 (34.7)	188,766 (32.3)	1202 (29.5)
Men		29,319 (63.2)	7360 (65.3)	395,949 (67.7)	2870 (70.5)
**Age groups (years)**					
0–9		7602 (17.1)	585 (5.3)	48,864 (8.4)	107 (2.6)
10–19		8544 (19.2)	1044 (9.4)	102,452 (17.5)	533 (13.0)
20–29		9215 (20.7)	3459 (31.1)	179,653 (30.7)	1608 (39.5)
30–39		7933 (17.9)	2953 (26.6)	123,801 (21.2)	1020 (25.1)
40–50		5621 (12.7)	1899 (17.1)	79,736 (13.6)	560 (13.8)
50 +		5485 (12.4)	1172 (10.6)	50,180 (8.6)	244 (6.0)
**Occupation**					
AD		12,947 (30.4)	624 (5.6)	24,392 (4.2)	23 (0.7)
TF		3515 (8.2)	391 (3.5)	147,035 (25.2)	1112 (27.3)
Mining		9336 (21.9)	8689 (78.1)	297,170 (50.8)	2564 (63.0)
Others		16,840 (39.5)	1418 (12.8)	122,188 (19.8)	366 (9.0)
**Race (indigenous)**					
No		33,320 (71.8)	10,607 (94.1)	548,367 (93.8)	3696 (90.8)
Yes		13,100 (28.2)	663 (5.9)	36,348 (6.2)	376 (9.2)
**Year**					
2016		8969 (19.3)	3254 (28.9)	152,037 (26.0)	825 (20.3)
2017		14,082 (30.3)	2912 (25.8)	226,196 (38.7)	1787 (43.9)
2018		23,369 (50.3)	5104 (45.3)	206,482 (35.3)	1460 (35.8)
**Species**					
PV		41,441 (89.3)	7796 (69.2)	451,261 (77.2)	2953 (72.5)
PF		4979 (10.7)	3474 (30.8)	133,450 (22.8)	1118 (27.5)
**Country of infection**					
Brazil		35,150 (75.7)	0 (0.0)	51 (0.01)	50 (1.2)
Venezuela		9270 (20.0)	9270 (82.3)	580,625 (99.3)	0 (0.0)
Guyana		1957 (4.2)	1957 (17.4)	4031 (0.7)	4016 (98.9)
Other countries		43 (0.1)	43 (0.4)	8 (0.0)	6 (0.2)

*PV* *Plasmodium vivax*, *PF* *Plasmodium falciparum, AD* agriculture and domestic, *TF* tourism, fishing and timber, *others* other occupations.

### Time-series decompositions

**Figure 2:**
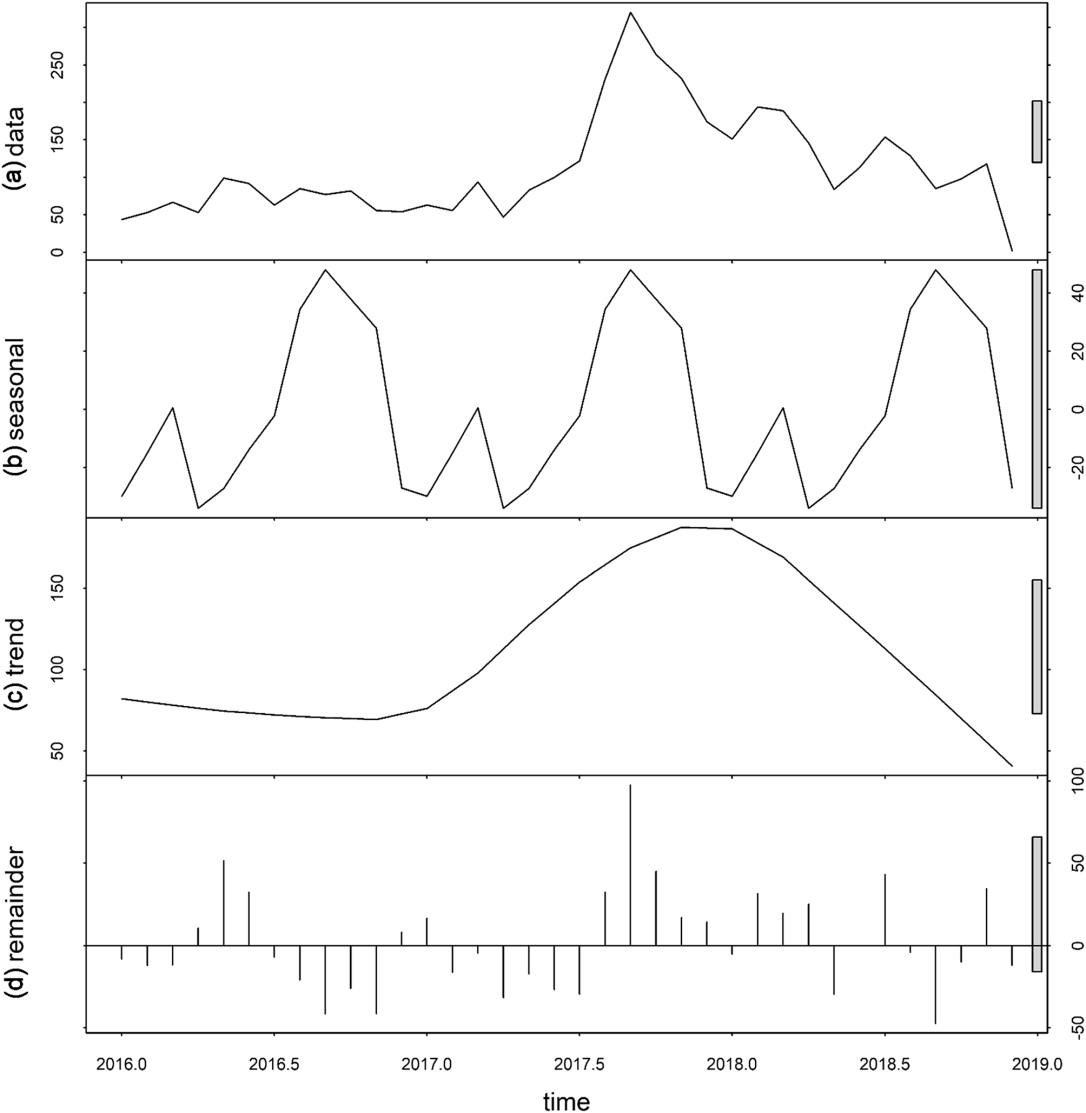
Temporal decomposition of imported malaria in Roraima, Brazil, 2016–2018. **(a)** The original time series, **(b)** the decomposed components, denoting the seasonal component, **(c)** long-term trend component and (**d**) remainder component.

**Figure 3:**
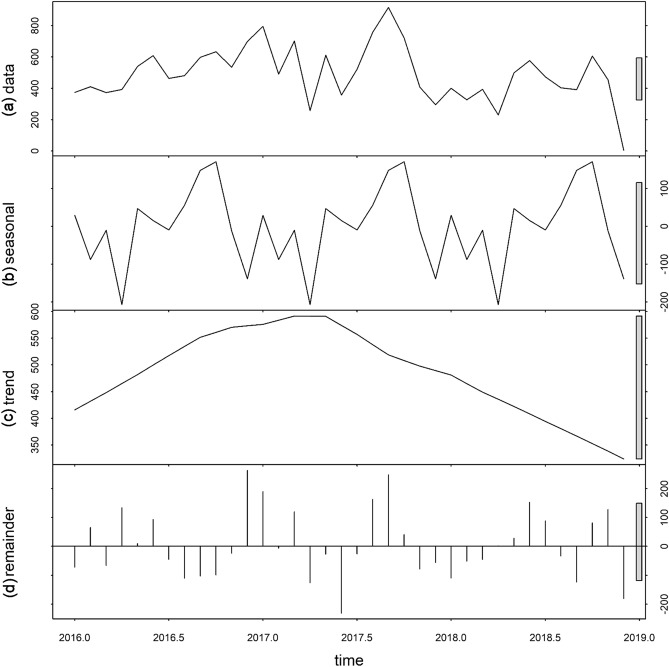
Temporal decomposition of imported malaria in Bolivar, Venezuela, 2016–2018. **(a)** The original time series, **(b)** the decomposed components, denoting the seasonal component, **(c)** long-term trend component and **(d)** remainder component.

In Roraima, the time-series decompositions of raw data showed a clear seasonal pattern in imported cases. Two peaks occured in the first half of the year, with incidence dropping off toward the middle of the year (June). The inter-annual pattern showed a large peak in 2018 with the lowest number of cases in the early part of 2017 (January–March) (Fig. 2 and Supplementary Fig. 1). For Bolivar, the seasonal pattern was irregular, with a large spike around August and September of each year. The trend showed a gradual increase in imported cases with a large peak in September 2017, steadily decreasing towards the end of 2018 (Fig. 3 and Supplementary Fig. 2).

### Spatial autocorrelation analysis

**Figure 4:**
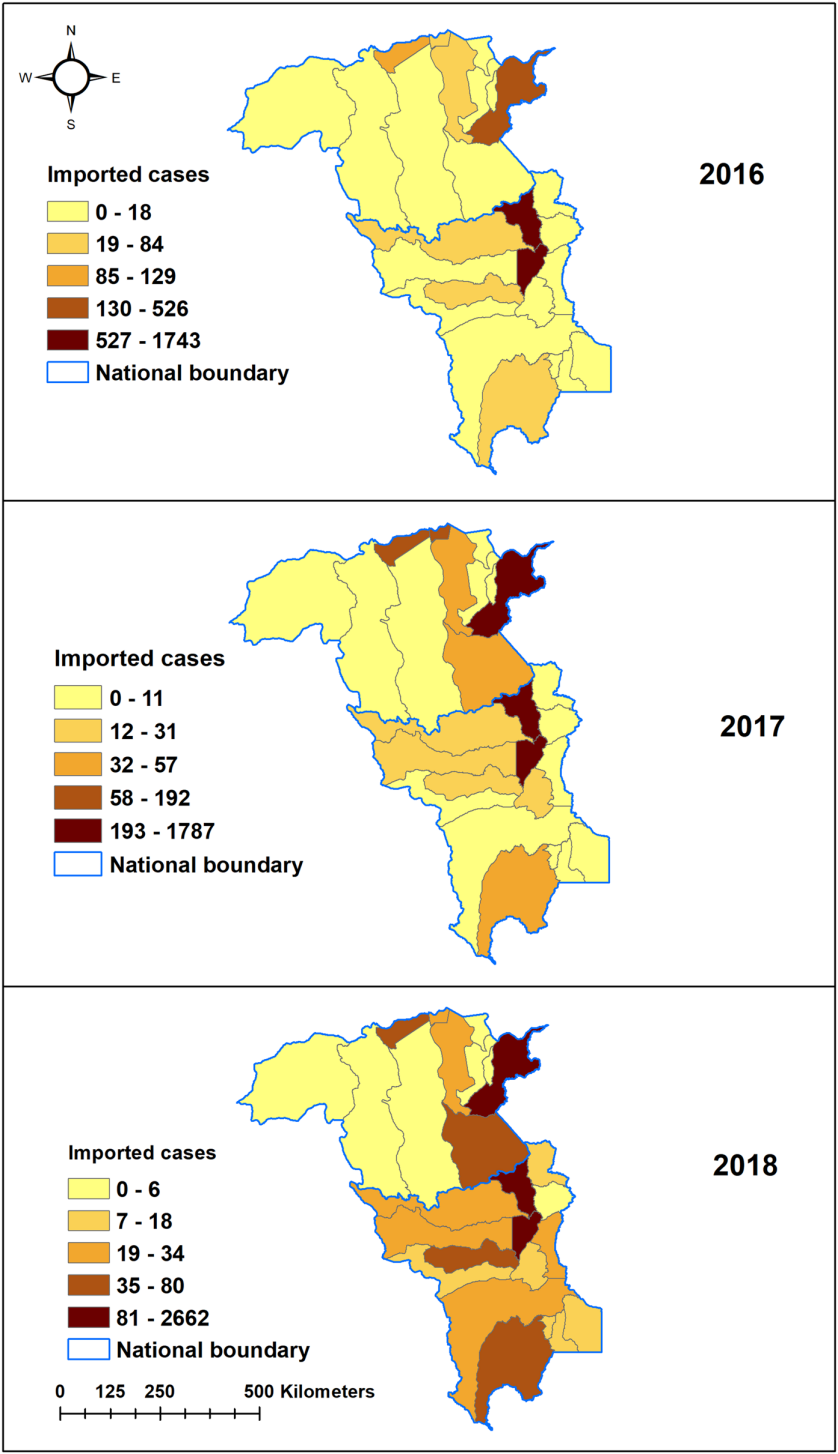
Imported malaria cases by municipalities in Roraima and Bolivar states, 2016–2018.

**Figure 5:**
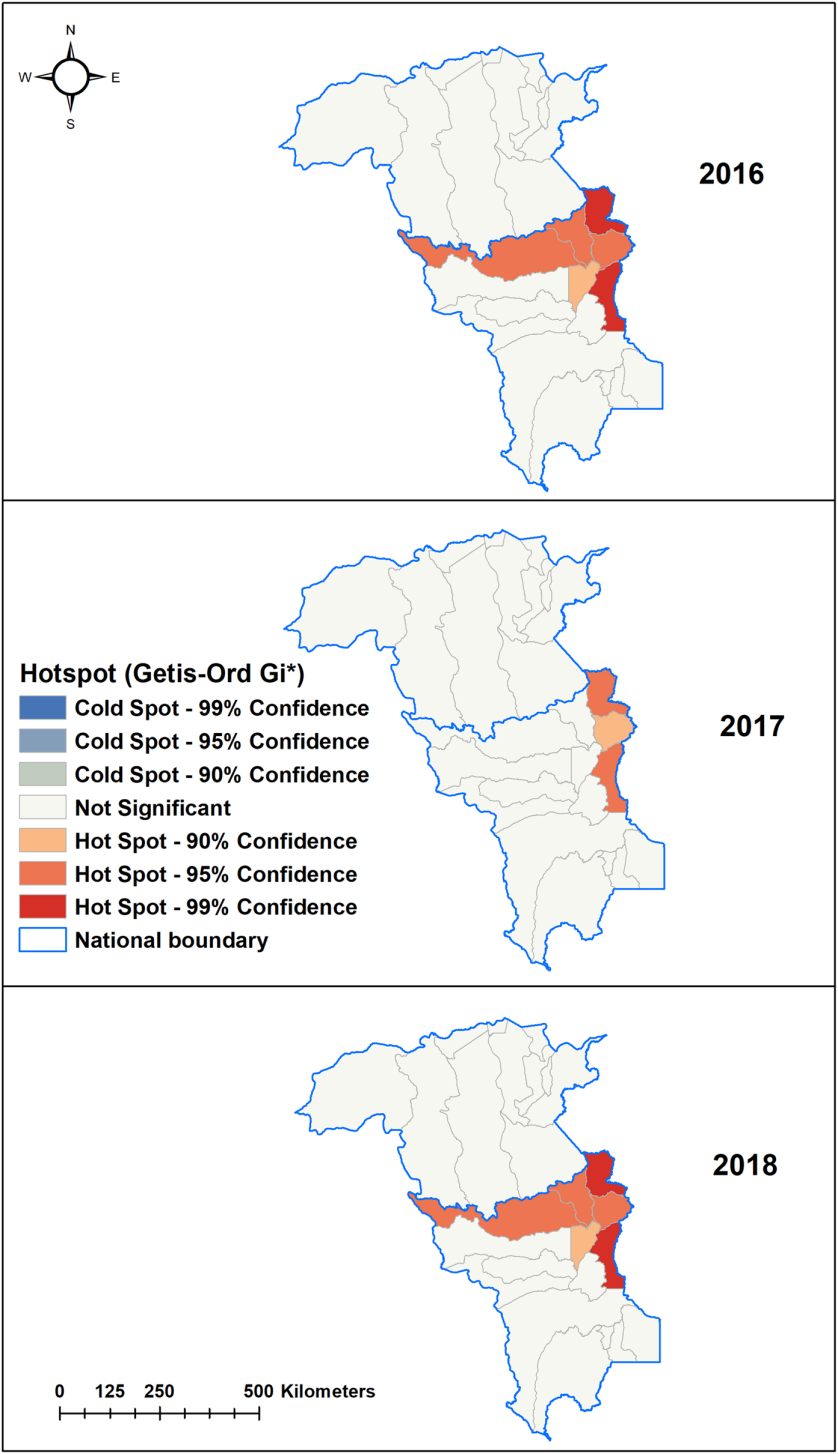
Hot spots (Getis-Ord Gi*) of imported malaria cases by municipalities in Roraima and Bolivar states, 2016–2018.

In 2016, Pacaraima and Boa Vista municipalities of Roraima state reported higher numbers of imported cases (range 527–1743 cases) and Sifontes municipality in Bolivar (range 130–526 cases) as compared to other municipalities in these states. This trend continued in 2017 in Roraima state, while cases increased to 58–192 cases in Caroni and Heres municipalities in 2017 in Bolivar state. However, in 2018, two more municipalities in Roraima namely Mucajai and Rorainopolis municipalities (range 35–80 cases), in addition to Pacaraima and Boa Vista, reported increased imported malaria cases. Similarly, in 2018, in addition to Sifontes and Heres municipalities in Bolivar, Gran Sabana municipality reported higher cases (range 35–80 cases) than other municipalities (Fig. 4). Hot spots were identified in six municipalities (Amajari, Pacaraima, Uiramuta, Normandia, Boa Vista, Bonfim) in the north-eastern part of Roraima in 2016 and 2018. In 2017, hotspots were limited to Uiramuta, Normandia, and Bonfim municipalities of Roraima state, located in the eastern part of the state near the border with Guyana (Fig. 5).

### Risk factor analysis

Results from the multivariable logistic regression showed that in Roraima, men were 28% less likely to be an imported case as compared to women (Adjusted Odds Ratio [AOR] = 0.72; 95% confidence interval [CI] 0.665, 0.781). Ages 20–29, 30–39 and 40–49 were more likely to be an imported case compared to the 0–9 year age group: 90% more likely in the aged 20–29 year group (AOR = 1.90; 95% CI 1.649, 2.181); 54% more likely in the 30–39 year group (AOR = 1.54; 95% CI 1.331, 1.782), and 33% greater odds in the 40–49 year group (AOR = 1.33; 95% CI 1.132, 1.552). Imported cases were 197 times (AOR = 197.03; 95% CI 175.094, 221.712) more likely to be in miners as compared to those working in the agriculture/domestic worker sector, which was the reference group. Similarly, occupations including tourism, timber and fishing and other occupations were about three (AOR = 2.99; 95% CI 1.591, 3.442) and two times (AOR = 2.05; 95% CI 1.853, 2.272) more likely to be imported cases, respectively. Indigenous people were 76% less likely to be an imported case (AOR = 0.24, 95% CI 0.213, 0.274) compared to non-indigenous people. *P. falciparum* had higher odds of being imported cases compared to *P. vivax* (AOR = 5.96, 95% CI 5.324, 6.667).

In Bolivar, all age groups: 10–19 years (AOR = 1.75; 95% CI 1.398, 2.192), 20–29 years (AOR = 2.48; 95% CI 1.957, 3.144), 30–39 years (AOR = 2.29; 95% CI 1.803, 2.913), 40–49 years (AOR = 1.97; 95% CI 1.542, 2.523) and > 49 years (AOR = 1.45, 95% CI 1.112, 1.882), were at higher risk of being an imported case compared to the 0–9-year-old group. Compared to those working in agriculture and domestic areas, those engaging in tourism, timber and fishing (AOR = 6.38; 95% CI 4.393, 9.254) and the mining sector (AOR = 7.03; 95% CI 4.903, 10.092) were seven times more likely to be an imported case. Similarly, “other” occupations were over three times more likely to be an imported case (AOR = 3.50; 95% CI 2.388, 5.139). Odds of being an imported case were nearly 51% (AOR = 0.49, 95% CI 0.438, 0.544) lower in indigenous people compared to non-indigenous people. *P. falciparum* cases had 21% (AOR = 1.21; 95% CI 1.129, 1.297) higher odds of being an imported case than *P. vivax* cases (Table 2). In contrast to Roraima, in Bolivar, the odds of being an imported case did not differ by sex of the case (AOR = 1.06; 95% CI 0.951, 1.181).

**Table 2:** Multivariable logistic regression of imported malaria in Roraima, Brazil and Bolivar, Venezuela: 2016–2018.

Characteristics		Roraima, Brazil			Bolivar, Venezuela		
		AOR	95% CI	*P* value	AOR	95% CI	*P* value
**Sex**							
Women		Ref			Ref		
Men		0.72	0.665, 0.781	< 0.001	1.06	0.951, 1.181	0.296
**Age groups (years)**							
0–9		Ref			Ref		
10–19		0.83	0.713, 0.968	0.017	1.75	1.389, 2.192	< 0.001
20–29		1.90	1.649, 2.181	< 0.001	2.48	1.957, 3.144	< 0.001
30–39		1.54	1.331, 1.782	< 0.001	2.29	1.803, 2.913	< 0.001
40–49		1.33	1.132, 1.552	< 0.001	1.97	1.542, 2.523	< 0.001
50 +		0.00	0.679, 0.949	0.01	1.45	1.112, 1.882	0.002
**Occupation**							
AD		Ref			Ref		
TF		2.99	2.591, 3.442	< 0.001	6.38	4.393, 9.254	< 0.001
Mining		197.03	175.094, 221.712	< 0.001	7.03	4.903, 10.092	< 0.001
Others		2.05	1.853, 2.272	< 0.001	3.50	2.388, 5.139	< 0.001
**Race (indigenous)**							
No		Ref			Ref		
Yes		0.24	0.213, 0.274	< 0.001	0.49	0.438, 0.544	< 0.001
**Species**							
PV		Ref			Ref		
PF		5.96	5.324, 6.667	< 0.001	1.21	1.129, 1.297	< 0.001

*Ref* reference group, *PV* *Plasmodium vivax*, *PF* *Plasmodium falciparum.*

*AOR* adjusted odds ratio, *CI* confidence interval, p-value < 0.05.

*AD* agriculture and domestic, *TF* tourism, fishing and timber, *Others* other occupations.

In Roraima, the crude OR between sex and imported malaria was 1.13 (95% CI 1.08, 1.18); when stratified by occupation, the stratified estimates were protective in some subgroups while increasing risk in another- 0.55 for mining and 1.22 for “other” occupations. The homogeneity test for interaction between sex and occupation on imported malaria status was *p* < 0.0001, indicating strong evidence of an interaction, meaning that the effect of sex on imported malaria is modified by occupation. The likelihood ratio test showed that the model with the interaction term was a better fit (p < 0.0001). The results of the logistic regression model with the interaction term between sex and occupation are shown in Supplementary Table 1, stratified by sex and occupational category.

Being male is protective from imported malaria in three out of four employment categories, and most protective in the agriculture/domestic sector, where men are 51% less likely to be an imported case than women (95% CI 0.42, 0.58; p < 0.0001). In the category denominated as “other,” the odds of being an imported case do not differ between males and females (AOR = 0.97, 95% CI 0.86, 1.09, p = 0.58).

Stratified by employment sector reported at time of diagnosis, females in each sector had lower odds of being an imported case than their male counterparts when compared to the reference category (agriculture/domestic work). The AOR for risk of being an imported case for women in mining was 197.68 (95% CI 160.47, 243.52) versus 218.02 for men (95% CI 187.57, 253.43).

## Discussion

This study aimed to describe the spatial and temporal epidemiology of imported malaria and its risk factors in Roraima, Brazil and Bolivar, Venezuela using secondary data from the two states from 2016 to 2018. In Roraima, females, cases aged 20–49 compared to those < 10 years, miners and ‘other’ occupations were more likely to report being an imported malaria case compared to those working in agriculture and domestic sectors. This was similar to Bolivar, except that, in addition to the occupations mentioned above in Roraima, those engaging in tourism and construction were also more likely to be an imported case. Non-indigenous people and *P. falciparum* cases were more likely to report imported malaria in both states. Spatially, hotspots of imported malaria were reported from the north-east municipalities of Roraima throughout the study period.

Similar to the study by Arisco et al., after adjusting using logistic regression, women were at higher risk of imported malaria in Roraima state^17^. This finding is different from other studies that reported being male as a risk factor for imported malaria^28,29^. This finding could be suggestive of an epidemiological shift from men to women, who may be increasingly engaging in mining and other occupations with high risk of malaria transmission. Other plausible reasons could be demographic shifts in migration patterns, from migration of predominantly men to family units with both genders. Therefore, further studies are required to understand this finding. However, analysis for interaction between sex and occupation showed that occupation was an effect modifier of the effect of sex on being an imported case, with the effects differing by occupational category.

Additionally, being a miner was the main risk factor of imported malaria in both Roraima and Bolivar states. Miners are at an increased risk of malaria infection due to prolonged exposure when working at mining sites (mining deforestation associated), which promotes habitats for *Anopheles* vectors and heightened host-vector contact extending up to several kilometers^30–33^. Increased surveillance, diagnosis, and treatment will remain the key prevention and control measures in the study area due to exophagic, exophilic and early biters^34^. However, extension of control and preventive measures such as (long lasting insecticidal nets [LLINs] and long-lasting insecticidal hammocks [LLIHs]) to this at-risk group should be used as adjuncts to achieving malaria control and elimination.

In both areas, the 20–49 age group was the most at risk of being an imported malaria case. Similar findings were reported in other studies^17^. This could be linked to migration, since most migrant populations that come to Roraima are in these groups (20 and 39 years)^17^. These younger adults are more likely to be involved in occupations such as mining and have more freedom to leave their homes to migrate to other states or countries to look for work, thereby increasing risk of imported malaria infection.

*Plasmodium falciparum* cases were more likely to be imported than *P. vivax* in both states*.* This could be a reflection of imported malaria associated with working in mining areas, since other studies have shown that *P. falciparum* was predominant in several gold mining regions, even though *P. vivax* consists of 75% of malaria cases in the Americas^4,35^. Imported malaria was predominantly reported in non-indigenous population in both the states. Similar findings have been reported in other studies^17^, primarily because non-indigenous people are involved in gold mining more than the indigenous. In Roraima, only 4.7% of cases in indigenous people were reported their occupation as mining compared to 28.6% on non-indigenous people. Similarly, 27.7% versus 52.4% worked in mining in Bolivar.

Spatial analysis revealed hotspots of imported malaria reported in the north-eastern part of Roraima bordering Bolivar and Guyana throughout the study period. This could be due to the location of illegal mining areas in these hotspot municipalities or because these municipalities are the central route of migration between the countries^28^. Lover et al. reported that many Brazilian miners travelled to Guyana to work in mining and returned to Boa Vista for medical care^21^. This is a plausible occurrence in this study, with Boa Vista municipality in Roraima reporting 55% (6192) of imported cases during the study period (Table 1). Increasing health services including malaria clinics in this area will reduce self-medication with under-the-counter antimalarials which are frequently reported in gold miners, though not legal in Brazil^36^. Distribution of preventive measures such as LLINs, LLIHs, and health education in these hotspot municipalities can help reduce malaria transmission in these groups. An earlier study reported Brazilian miners did not know how malaria was transmitted and associated malaria infection with contaminated water and food^37^. In addition, early diagnosis and treatment should be strengthened in mining areas, along with improved regulation of mining activities, which can together make a significant reduction in importation of malaria.

The results of this study need to be interpreted considering some limitations. First, the major limitation of this study is the use of surveillance data. As such, completeness and representativeness of such data could not be fully ascertained. Second, malaria self-diagnosis or diagnosis and treatment in private health settings could have been unaccounted for. However, in Brazil treatment outside the public sector is prohibited, though there is an informal market for malaria treatment in some municipalities of Roraima, mainly in remote and illegal mining sites. Self-medication is common among miners and could be a problem resulting in an underestimation of actual cases^37,38^. Third, important risk factors including education level could not be included in the analysis, since these data were not recorded in Bolivar state. Fourth, unmeasured risk modifiers, such as socio-economic development, living standards, treatment, localized behavioral patterns, population mobility, bed net use and residual indoor insecticide coverage were not included in this study, and could be included in future analysis. However, IRS can be difficult and inefficient when used in temporary shelters with provisional tents, thatches or corrugated iron rooms and plastic walls or local materials (tree branches). Fifth, malaria cases infected in Bolivar state but diagnosed and treated in other states of Venezuela were not accounted in this study.

Globally, a number of pilot studies have been undertaken to test and develop interventions aimed at cross-border populations. For instance, mobile malaria clinics have shown to be effective in increasing access to malaria services for hard-to-reach populations, such as miners^39–41^. Mobile teams can complement routine facility-based health services. A study in Myanmar showed that a mobile malaria clinic provided malaria services including diagnosis and testing, provision of control measures, and health education to more people and had wider geographical coverage than a community health center^39^. Mobile Malaria Workers (MMWs) have also been able to deliver quality malaria services to migrant populations (seasonal workers)^42^. For example, in Cambodia, they were very efficient in interpersonal communication and became the most trusted source of information. MMWs were also well received by the communities they serve and viewed as an economic advantage by the farm owners. MMWs were effective and reliable at providing both prevention, and early diagnosis and treatment to seasonal migrant workers.

In addition, screening posts could serve as important service points for mobile populations^5^. These posts can be set up at strategic locations including border crossings and migration portals: taxi stands, and public bus and boat terminals. They can be used to provide intervention packages such as LLINs and LLIHs, pamphlets on signs and symptoms of malaria, and can serve as possible contact points for malaria diagnosis and treatment services in the destination area, and education on malaria prevention^5^.

Furthermore, free screening and treatment for asymptomatic malaria can be offered for both returning and travelling migrants at the posts^43^. The posts can also serve as surveillance centers to collect information on the destination of travelers and inform relevant public health officials in destination areas. Additionally, treatment of asymptomatic cases can reduce sources of infection from fellow workers or travelers at the destination^12^. However, most current malaria control programs are not integrating approaches such as MMWs or clinics and screening posts through the implementation of outreach clinics in remote areas, or targeting hard-to-access population groups as part of routine services^12^. Another novel approach to improving diagnosis and adherence to treatment among informal miners has been used in French Guiana, where Malakit targets gold miners working illegally with free malaria self-diagnosis and self-treatment kits for *P. falciparum*^44^. Furthermore, it is critical that health planners and policymakers support and fund the expansion of such novel approaches in the drive to eliminate malaria and tailor malaria control and treatment approaches as transmission dynamics change quickly over time due to external and internal factors^45^.

In order to better understand the mobility dynamics, profiles of different groups, their demographics, and malaria prevention and treatment-seeking behaviour practices, we recommend conducting respondent-driven sampling (RDS) studies among these populations^26,27,46^. The RDS methodology is a modified form of snowball sampling that can be used to recruit groups that do not congregate in stable and identifiable places^47^. It is suitable for cross-border populations as it was developed as a method of achieving reliable, statistically robust estimates for populations for whom sampling frames may be impossible to generate^48^.

Moreover, regional initiatives that focus on strengthening regional coordination to achieve malaria elimination are critical to addressing challenges to elimination^21^. In the Americas, the major regional initiatives are: the Amazon Malaria Initiative (AMI), the Amazon Network for the Surveillance of Antimalarial Drug Resistance (RAVREDA)^49^. Other relevant regional initiatives in other parts of the world are the Asia Pacific Malaria Elimination Network (APMEN)^10^, the Asia Pacific Leaders Malaria Alliance (APLMA) , the African Leaders Malaria Alliance (ALMA), and The Elimination Eight Initiative (E8)^50^.

## Conclusion

Our results have shown that cross-border populations need targeted and tailored intervention strategies according to their occupation, age and mobility patterns, focusing in hotspot areas. Furthermore, all stakeholders, including implementers, policymakers and donors, should support and explore the introduction of novel approaches to address these hard-to-reach populations.

## Methods

### Study area and data

The study area included the state of Roraima state, Brazil, and Bolivar state, Venezuela (Fig. 1). Roraima and Bolivar are divided into 15 and 11 municipalities, respectively. Individual-level de-identified datasets were obtained from the Brazilian Malaria Epidemiology Surveillance Information System (SIVEP-Malaria), the Venezuelan Ministry of Health, and Venezuelan civil society organizations. Individual-specific data extracted were age, sex, occupation, date of diagnosis and treatment, malaria species, race (indigenous or non-indigenous) and type of malaria transmission (imported or autochthonous). Imported malaria was defined as those cases infected in a country other than the country where the cases presented or notified, whereas autochthonous malaria (referred to as indigenous case) was defined as cases infected in Roraima and Bolivar states, or other states in the same country.

Populations of the states and municipalities were obtained from projections based on 2010 and 2011 census data in Brazil and in Venezuela, respectively^51^. An electronic map of municipalities in shapefile format was obtained from the DIVA-GIS database (https://www.diva-gis.org/).

### Exploration of seasonal patterns and inter-annual patterns

The average monthly number of imported malaria cases was calculated from the full time series (January 2016–December 2018). The time series of malaria incidence was decomposed using seasonal-trend decomposition based on locally (STL) weighted regression to show: the seasonal pattern, inter-annual patterns and the residual variability. The STL model was structured as follows:

$${Y}_{t}={S}_{t}+{T}_{t}+{R}_{t}$$

where *Y*_*t*_ represents numbers of imported malaria cases with logarithmic transformation, *S*_*t*_ is the additive seasonal component, *T*_*t*_ is the trend, and *R*_*t*_ is the “remainder component”; *t* is time in months^52,53^.

### Hotspot analysis

Yearly cumulative imported cases were used for hotspot analysis. We ran both Anselin Local Moran’s I and Getis-Ord statistic (Gi*), and Gi* statistic provided a better fit for these data. The presence and nature of spatial autocorrelation that suggest malaria clustering were assessed by the Getis-Ord statistic (Gi*)^54,55^. The local Getis-Ord statistic (Gi*) was used to identify the intensity and stability of hotspot/coldspot clusters^55,56^. The Gi* statistic compares the mean rate of malaria by location of infection (i.e., the rates of malaria for a target location and its neighbors) to the global malaria mean rate (the rates of all municipalities). The Gi* statistic compares the z-score and p-value for each municipality and with global malaria means. Locations with a statistically significant and larger z-score will have a more intense clustering of high values (hotspots), where it is very unlikely that the spatial clustering of high values is the result of a random spatial process. Locations with a statistically significant and smaller z-score will have more intense clustering of low values (coldspots)^55^. We used a fixed distance band for spatial relationships. Malaria in each municipality was analyzed within the context of neighboring municipalities. Neighboring municipalities inside the specified critical distance (Distance Band or Threshold Distance) receive a weight of one and exert influence on computations for the target municipality, while those municipalities outside the critical distance receive a weight of zero and have no influence on a target feature's computations. We did not specify any distance band or threshold and used the default setting of the software. ArcMap 10.5.1 software (ESRI, Redlands, CA) was used for hotspot analysis and creating maps.

### Statistical analysis

Univariate and multivariable logistic regression models were built using backward elimination for imported malaria compared to autochthonous malaria to identify significant covariates. Any variable with a *p*-value < 0.2 in the univariate analysis, along with the main variable of interest, was considered as a candidate variable in the multivariable model. All potential dependent variables were entered in the full model, and adjusted odds ratios with 95% confidence intervals (CI) were used to determine the correlates of each independent variable. To test if the effect of sex on imported malaria status was modified by occupation, we first adjusted and stratified the association by occupation using Mantel–Haenszel methods, comparing the adjusted OR to the crude OR. The chi-square test of homogeneity determined if there was evidence of effect modification between sex and occupation, and this was further tested through the likelihood ratio test, which assessed if the logistic regression model was a better fit with or without the interaction term between sex and occupation.

Any variables that were ordinal in nature were tested for linear trends in the final regression model. All explanatory variables in the multivariable model were tested to ensure that there was no multi-collinearity in the final model using a variance inflation factor (VIF). VIF < 10 was considered a good fit of regression analysis. The analysis was performed using the statistical package Stata version 15 (Stata Corporation, College Station, TX, USA).

### Ethical considerations

The research protocol was approved by the National Center of Bioethics in Venezuela (CENABI). The National Survey Ethics Council (CONEP) deemed that ethical clearance for the use of this secondary data in Brazil was not necessary. The malaria database was obtained through e-sic in Brazil^57^. All methods were performed in accordance with the relevant guidelines and regulations. All participating researchers declared no conflict of interests.

### Ethics approval and consent to participate

The National Center of Bioethics in Venezuela (CENABI) approved the research protocol; the National Survey Ethics Council (CONEP) in Brazil considered that ethical clearance for the use of this secondary data was not necessary. Human participants were not involved in the study.

## Supplementary Information

Supplementary Information.

## Data Availability

The study dataset can be made available only upon the approval of researchers and organizations involved.
