# Supplementary material for: Cross-border malaria drivers and risk factors on the Brazil–Venezuela border between 2016 and 2018
Source: Sci Rep. 2022 Apr 11;12:6058. doi: 10.1038/s41598-022-09819-0 (PMC9001644; doi:10.1038/s41598-022-09819-0)
Supplement: Supplementary file 1 — Supplementary Information. [file 41598_2022_9819_MOESM1_ESM.pdf]

Cross-border malaria drivers and risk factors on the Brazil-Venezuela border between 2016 and 2018

Kinley Wangdi[1*¥,] Erica Wetzler[2¥], Paola Marchesini[3], Leopoldo Villegas[4,5], Sara Canavati[2]

[1]Department of Global Health, Research School of Population Health, ANU College of Health and Medicine, The Australian National University, Canberra, ACT 2601, Australia

[2]World Vision US, 34834 Weyerhaeuser Way South, Federal Way, Washington, USA

[3]Department of Surveillance for Zoonotic and Vector Borne Diseases, Malaria Technical Group, Ministry of Health, Brasilia, Federal District, Brazil

[4]Global Development One, Silver Spring, Maryland, USA

[5]Asociación Civil Impacto Social (ASOCIS), Tumeremo, Bolívar, Venezuela

*Corresponding author

[¥]Contributed equally

Email:

KW: kinley.wangdi@anu.edu.au

EW: ewetzler@worldvision.org

PM: paola.b.marchesini@gmail.com

LV: leopoldovillegas2@gmail.com

SC: saracanavati@yahoo.com

Supplementary Material

Supplementary Table 1 Multivariable logistic regression for the effect of various risk factors on imported malaria, with an interaction term between sex and occupation, Roraima state Brazil, 2016 to 2018

**Characteristics**		**AOR**	**95% CI**	**P-value**
Odds of imported malaria by sex among different occupational groups				
Agriculture/domestic	Female	1.00		
	Male	0.49	0.42, 0.58	<0.0001
Timber fishing	Female	1.00		
	Male	0.75	0.59, 0.97	0.021
Mining	Female	1.00		
	Male	0.54	0.45, 0.66	<0.0001
Other	Female	1.00		
	Male	0.97	0.86, 1.09	0.58
Odds of imported malaria by occupation among men and women				
Female	Agric/domestic	1.00		
	Timber/fishing	2.35	1.87, 2.96	<0.0001
Male	Agric/domestic	1.00		
	Timber/fishing	3.60	3.00, 4.32	<0.0001
Female	Agric/domestic	1.00		
	Mining	197.68	160.47, 243.52	<0.0001
Male	Agric/domestic	1.00		
	Mining	218.02	187.57, 253.43	<0.0001
Female	Agric/domestic	1.00		
	Other	1.97	1.60, 2.42	<0.0001
Male	Agric/domestic	1.00		
	Other	2.72	2.37, 3.12	<0.0001
**Odds of imported malaria by other risk factors in the model**				
Age category (in years)	0-9	1.00		
	10-19	0.83	0.71, 0.96	0.015
	20-29	1.87	1.63, 2.15	<0.0001
	30-39	1.51	1.31, 1.75	<0.0001
	40-49	1.30	1.11, 1.52	0.001
	50+ years	0.79	0.67, 0.93	0.006
Race	Non-indigenous	1.00		
	Indigenous	0.24	0.21, 0.28	<0.0001
Species of malaria	*P. vivax*	1.00		
	P. falciparum	5.95	5.31, 6.65	<0.0001

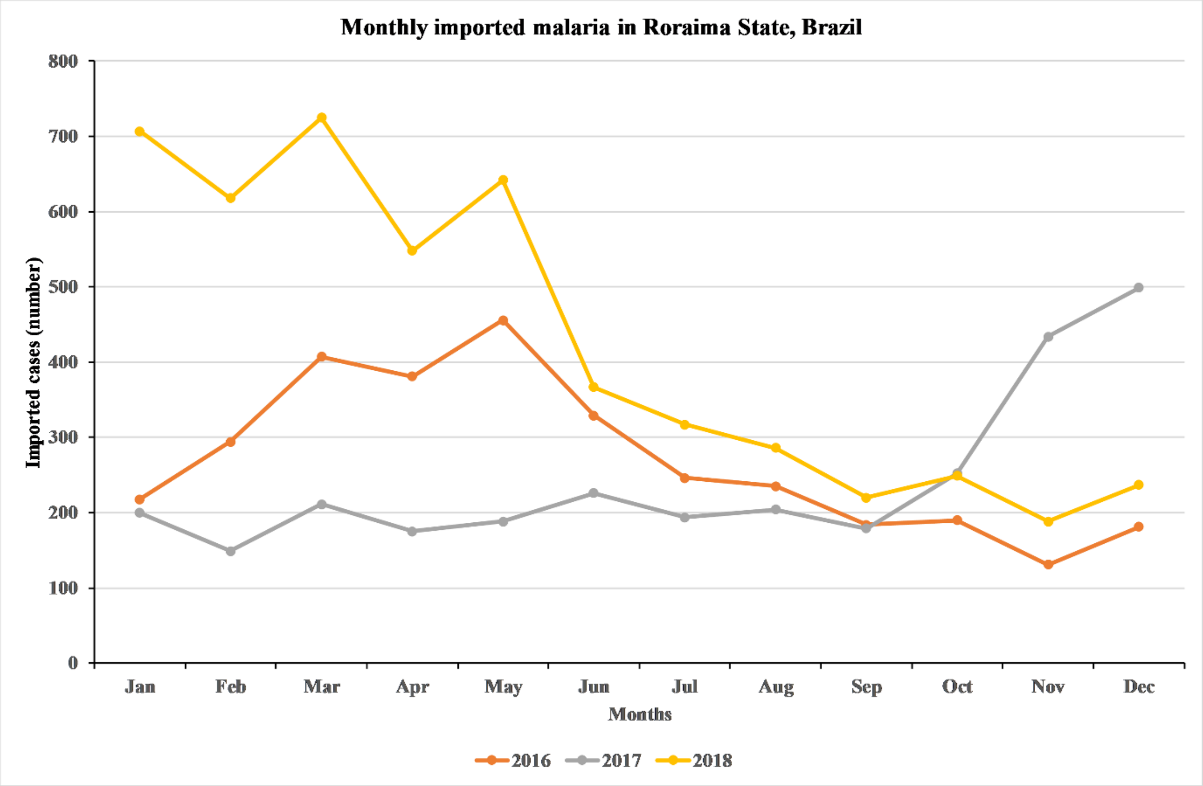

Supplementary Figure 1 Monthly trend of imported malaria in Roraima State, Brazil

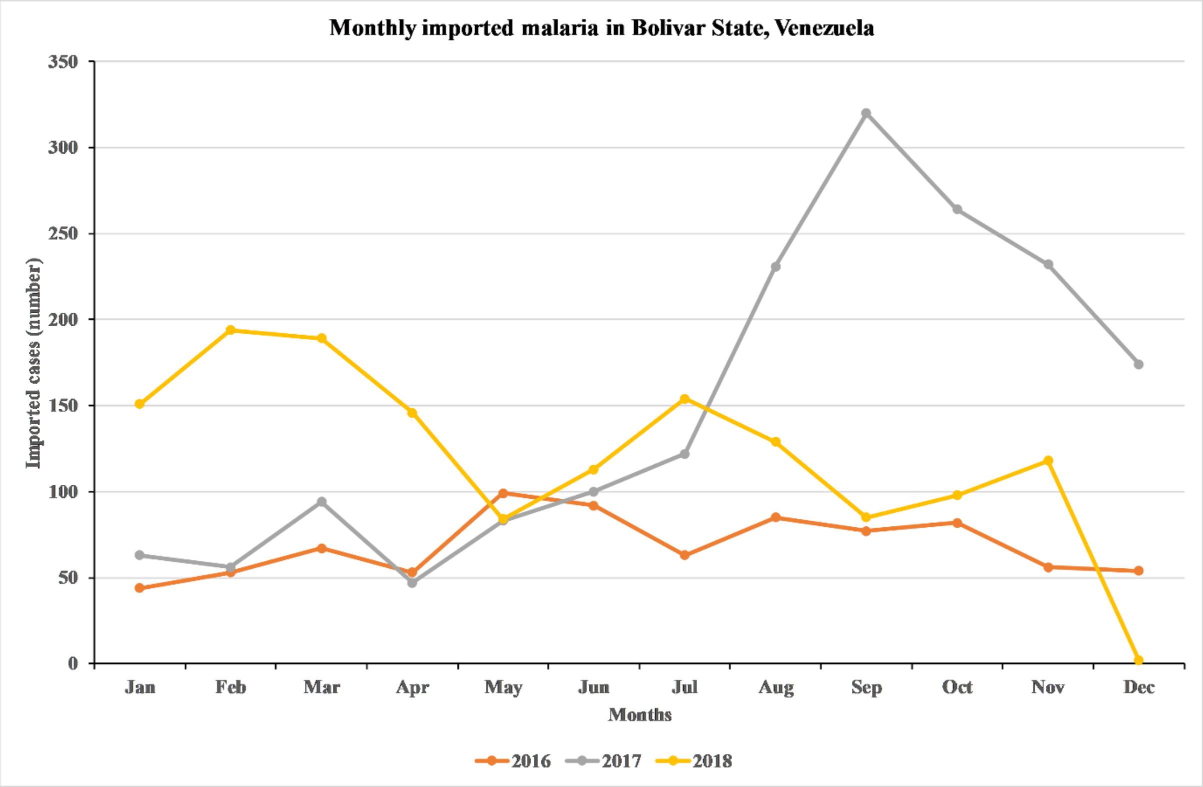

Supplementary Figure 2 Monthly trend of imported malaria in Bolivar State, Venezuela
